# Study protocol for a nested process evaluation of a complex discharge planning intervention (HOME Rehab) to improve participation after first-stroke

**DOI:** 10.3389/fneur.2024.1483245

**Published:** 2025-01-10

**Authors:** Sandra Reeder, Mandy Stanley, Laura J. Jolliffe, Natasha A. Lannin

**Affiliations:** ^1^Department of Neuroscience, School of Translational Medicine, Monash University, Melbourne, VIC, Australia; ^2^School of Medical and Health Sciences, Edith Cowan University, Joondalup, WA, Australia; ^3^Department of Occupational Therapy, School of Primary and Allied Health Care, Monash University, Melbourne, VIC, Australia; ^4^Allied Health, Peninsula Health, Melbourne, VIC, Australia; ^5^Department of Occupational Therapy, Alfred Health, Melbourne, VIC, Australia

**Keywords:** stroke rehabilitation, discharge planning, rehabilitation, occupational therapy, clinical trials, qualitative

## Abstract

**Introduction:**

Stroke is a leading cause of adult disability, and the transition from hospital to home can be fraught with challenges. The HOME Rehab trial is designed to address if better health outcomes for stroke survivors can be achieved with a contextually relevant and tailored occupational therapy discharge planning and support intervention. Process evaluations inform clinical trial findings and future scale up, as well as how to implement a successful intervention effectively into policy and practice. This paper describes the protocol we are using in the HOME Rehab process evaluation planning and activities.

**Methods:**

Using a theoretically informed approach, mixed methods are being used to collect data and address all aspects of the RE-AIM framework. Quantitative data will comprise clinician surveys, trial logs and fidelity checklists as well as screening and recruitment numbers. Semi-structured interviews with trial participants and carers and focus groups with occupational therapists will provide qualitative data. A concurrent triangulation approach will be taken to draw on the strengths of multiple methods to cross-validate findings. The RE-AIM framework will be used to interpret the qualitative and quantitative data together as well as highlight areas of convergence or divergence in the findings. Multiple data sources will be integrated to refine the interpretation of outcomes, understand the context of program delivery, and identify key findings. Drawing on, and integrating data from, multiple perspectives and methods will strengthen the overall findings and provided detailed insights into the causal mechanisms as well as the contextual factors that may influence intervention outcomes.

**Discussion:**

Process evaluations can optimize study outcomes by improving how a complex intervention is implemented, informing the actions of policymakers and clinicians. For the HOME Rehab intervention, the process evaluation may provide valuable data necessary to explain the trial findings, as well as inform future scale-up and implementation if the HOME Rehab intervention is shown to be effective.

**Clinical trial registration:**

https://www.anzctr.org.au, identifier ACTRN12618001360202.

## 1 Introduction

Globally, stroke is the leading cause of disability in adults and each year millions of stroke survivors must adapt to a life with restrictions in activities of daily living as a consequence (1). Stroke rehabilitation interventions, tested first in clinical trials and then translated into practice, reduce disability after stroke (2). Conducting process evaluations alongside clinical trials, and embedding theoretical frameworks within process evaluations, ensures trials effectively inform stroke policy and practice (3–5), providing vital evidence about how an intervention does or does not work, how an intervention is implemented, its mechanisms of impact, and the contextual factors that impact on the intervention. All of which provides details for scale up and replication in different settings should the program be effective (5).

The United Kingdom Medical Research Council (MRC) recommends that researchers conduct process evaluations alongside clinical trials (6). When implementing complex intervention, such as the HOME Rehab trial (7), process evaluations are particularly useful for understanding multiple interacting components, variable outcomes, and the new behaviors required by the people delivering or receiving the intervention (8). Key domains recommended to be evaluated in a process evaluation within the MRC guidance (context, quality of implementation and mechanisms of the intervention) are often augmented in process evaluations from established evaluation frameworks such as the Reach, Effectiveness, Adoption, Implementation and Maintenance framework (RE-AIM) (9). Together, these guidelines and frameworks seek to enable research translation from clinical trials to clinical practice.

### 1.1 About the HOME Rehab trial

The HOME Rehab trial is designed to address if better health outcomes for stroke survivors can be achieved with a contextually relevant and tailored occupational therapy discharge planning and support intervention; the full clinical trial protocol and intervention components has been published elsewhere (7). The HOME Rehab intervention was developed to address the known challenges experienced when transitioning from hospital to home after stroke, with some returning to hospital soon after discharge (10–12). Compared with transitions to nursing homes, transitions to home after stroke are associated with increased risk of readmission or emergency department visits (13), suggesting there are factors in the home environment which likely contribute to a patient’s early readmission. This context is perhaps complicated by the known insufficient communication and service coordination during discharge planning (14–16) with stroke survivors reporting they struggle with independence, social participation and resuming usual activities (17–20). With the numerous contextual factors and the complexity of the HOME Rehab trial (multiple sites, multiple states and health jurisdictions; interacting intervention components; collection of primary and secondary outcomes), conducting a process evaluation nested within the HOME Rehab trial will provide key evaluation data that will support interpretation of effectiveness data. This process evaluation will provide insights into how the intervention either worked or did not, explaining differences in outcome, and gain insights into the experience of both trial participants as well as clinicians working in rehabilitation.

### 1.2 Objective of the process evaluation

The overall aim of the process evaluation is therefore to explain the trial findings, as well as inform future scale-up and implementation if the HOME Rehab intervention is shown to be effective. If effective, understandings about implementation of the intervention, its mechanisms of impact, and contextual factors influencing delivery and functioning of the intervention will critically inform evidence-based stroke policy and practice (5).

Therefore, the specific objectives of the HOME Rehab Trial process evaluation are to:

- describe the characteristics of participating hospitals and participants to assess reach;
- explore the effects of individual intervention components on the primary outcome of participation;
- describe the perceived effectiveness of relevant intervention components [including the relationship between the participant and their occupational therapist, the importance of goals set, the in-hospital component (including the pre-discharge visit to the home), the community component (including the General Practitioner (GP) liaison), and staff training] from the participant, carer and occupational therapist perspectives;
- describe the perceived acceptance of the HOME rehabilitation intervention by involved occupational therapists and how their attitudes may or may not have shifted after training and involvement in the trial;
- outline the HOME Rehabilitation experimental intervention as delivered in terms of quality, quantity, adaptations and variations (planned and unplanned); and
- estimate the extent to which intervention delivery is normalized among the intervention healthcare professionals and related practice staff at the completion of the trial.

## 2 Methods and analysis

### 2.1 Design

**Figure 1:**
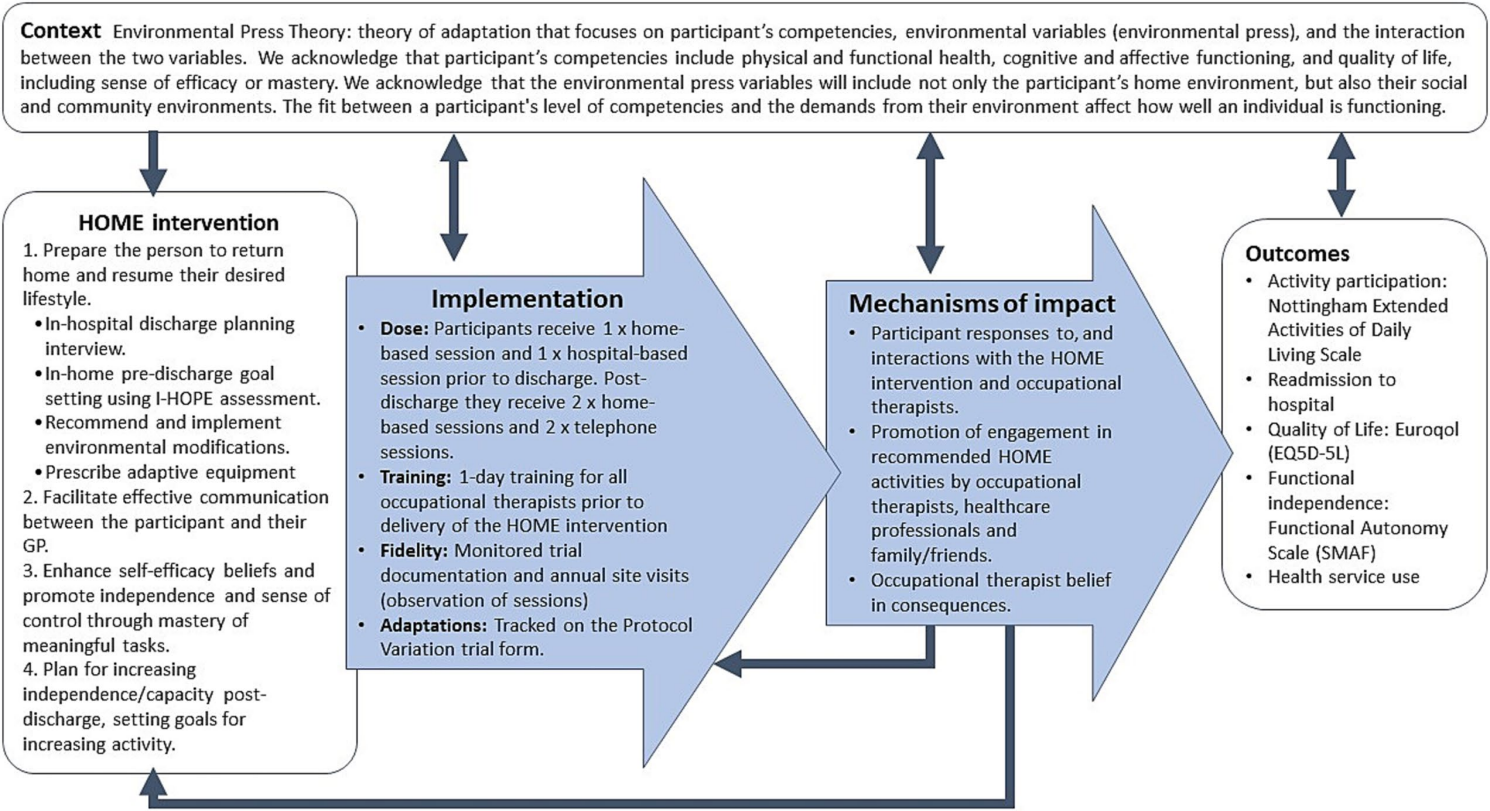
Key functions of the HOME trial process evaluation and relationships amongst them. Blue boxes represent components of process evaluation, which are informed by the causal assumptions of the HOME Rehab experimental intervention, and inform the interpretation of outcomes from the main trial.

The HOME Rehab trial is a multicenter, phase III RCT being conducted in Australia with concealed allocation, blinded measurement and intention-to-treat analysis (7). The setting is in-hospital rehabilitation centers across the states of New South Wales, Queensland, South Australia and Victoria; a list of trial sites is available on the trial registry. The MRC guidelines for process evaluation will provide an overall conceptual framework to evaluate the HOME Rehab trial (5), collating data on context (how context affects implementation and outcomes), implementation (what is implemented and outcomes) and mechanisms of the intervention (how the intervention produces change). These guidelines also emphasize the need to clarify the key causal assumptions made in developing the HOME Rehab intervention, which are outlined in Figure 1. Figure 1 shows these causal assumptions made, how they inform the key functions of the process evaluation, and how this will contribute to interpretation of outcomes from the main trial.

**Figure 2:**
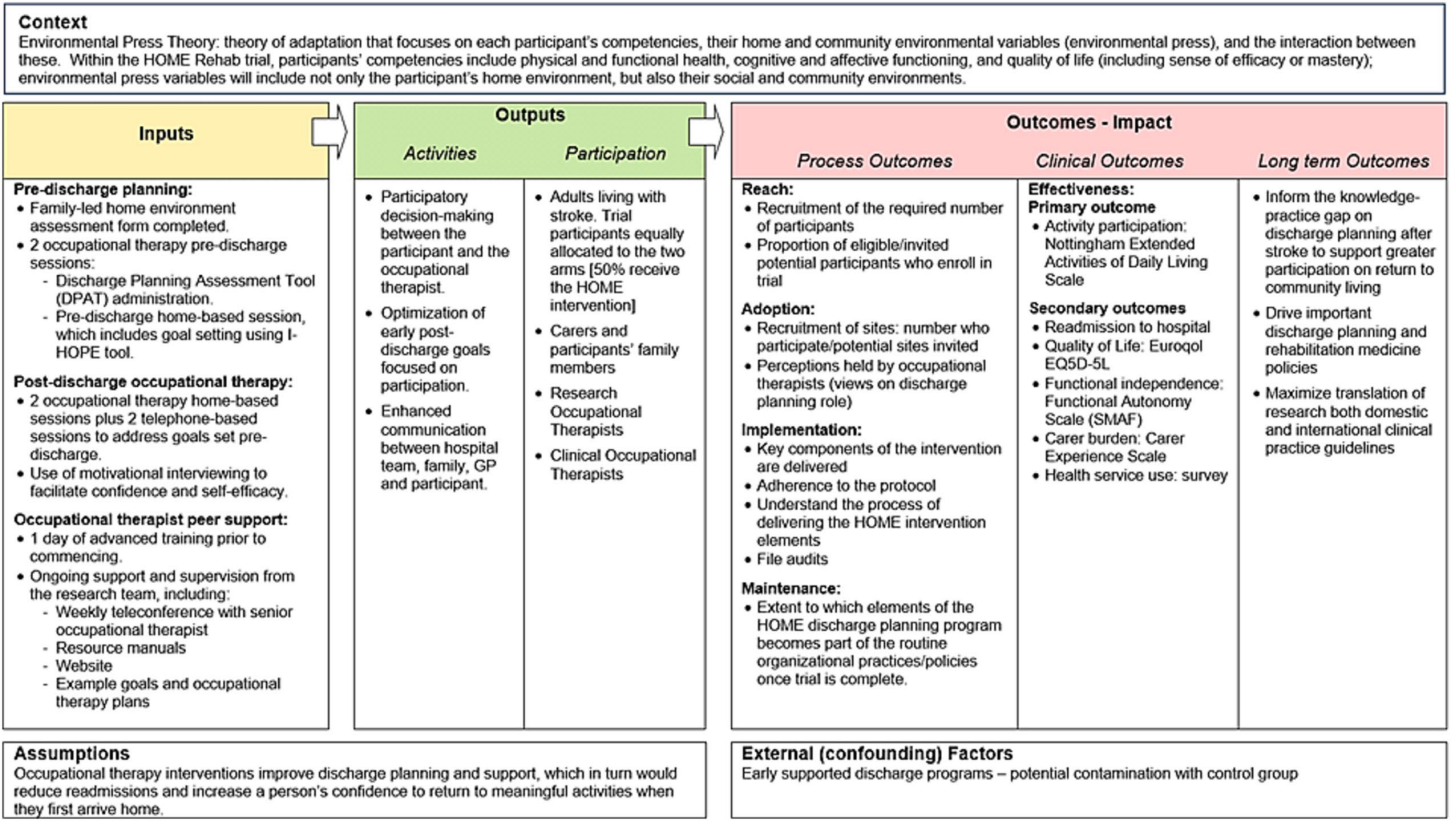
Logic model of the HOME trial process evaluation.

Within this mixed method process evaluation, the RE-AIM framework (9) will be applied to understand and describe the reach, effectiveness, adoption, implementation and maintenance of the HOME Rehab intervention. These five domains of the REAIM framework will enable a comprehensive, mixed-methods evaluation, one which will systematically explore implementation of the HOME Rehab intervention in the trial and allow the research team to prepare for ‘real world’ implementation as outlined in the logic model for the process evaluation (Figure 2).

### 2.2 Process evaluation study population

The process evaluation has been integrated into the randomised controlled trial (RCT) design of the HOME Rehab trial, and will therefore involve stroke clinicians (occupational therapists) working at participating centers, stroke survivors aged ≥45 years (HOME Rehab Trial participants), carers (of HOME Rehab Trial participants) and HOME Rehab trial occupational therapists.

### 2.3 Process evaluation methods

A mix of quantitative and qualitative approaches will therefore be used to address the aims of process evaluation (Table 1). A concurrent triangulation approach will be taken to draw on the strengths of multiple methods to cross-validate findings (21).

**Table 1:** Quantitative and qualitative methods of process evaluation data collection employed in the evaluation.

Stage of trial	Data collected
Pre-implementation	Records of engagement meetings with staff at participating sites Meetings with occupational therapy managers and rehabilitation physicians to understand service context and service models for discharge planning after first stroke Descriptions of the organizational characteristics of services using face-to-face interviews, observation and notes from site visits Survey of all occupational therapists (beliefs about occupational therapy discharge planning) Participant baseline data across primary and secondary outcomes
Recruitment	Site screening, eligibility and recruitment log (includes reasons for ineligibility or non-participation) to report participant recruitment and retention as per CONSORT. Site bed numbers and average length of stay to report representativeness of site Participant hospital length of stay, age, and stroke severity to report representativeness of participants
Implementation	Intervention provider details (includes expertise, background and specific training provided) to report against TIDieR checklist Fidelity monitoring checklists for all components of the intervention completed at participating sites for each intervention provider annually Diary of co-rehabilitation intervention information (including use of community services, hospital readmission and healthcare resource utilization data from time of discharge to end of 12 months) Intervention documentation per participant, including session number and length, mode of delivery, session goals, activities completed, equipment/materials provided and trial protocol variation records Costs of delivering the intervention Records of meetings with key staff across trial (site coordinators, occupational therapists, consumer engagement panel, trial management committee, data safety and monitoring committee)
Post-intervention	Participant end of intervention (4-weeks post discharge) and follow-up (6-months post discharge) across primary and secondary outcomes Post-intervention readmission and healthcare resource utilization, including pharmaceutical and cost data Caregiver burden at post-intervention (4-weeks post-discharge) and follow-up (6-months) Face-to-face or telephone interviews with participants, carers and occupational therapists who participated in the intervention Survey of all occupational therapists (beliefs about occupational therapy discharge planning) Organizational survey (12-months following final participant recruited) to report maintenance of trial intervention elements

CONSORT, Consolidated Standards of Reporting Trials; TIDieR, Template for Intervention Description and Replication.

To address the ‘reach’ REAIM dimension, the HOME process evaluation will collect data on the characteristics of participating hospitals and participants. The representativeness of participating hospitals and participants will be evaluated against Australian stroke statistics including hospital length of stay and bed numbers; participant age, and stroke severity. Further, we will maintain an audit of trial recruitment log to record the willingness of potential participants to participate in the study. This will provide information on the proportion of eligible/invited potential participants who enrol in trial. Reasons for potential participant’s non-participation will be included from each site’s log.

For the effectiveness dimension, the process evaluation will explore perceptions of the benefits of individual intervention components on the primary outcome of participation. Each intervention component has been outlined in the published protocol (7), allowing the interviewer to qualitatively explore perceived effectiveness of, for example, the pre-discharge visit separately to the goal setting component. To triangulate data and gain detailed understandings, semi-structured interviews with participants and carers, and focus groups with occupational therapists will explore the mechanisms through which the intervention brings change. Knowledge about the mechanism is vital for understanding how and why the intervention is or is not effective and for future replication.

To explore patient and carer perspectives, the trial coordinator will prepare a list of participants from the HOME Rehab trial who have completed their 4-week assessment but are within 12 months of discharge (noting demographics and attributes to facilitate maximum variation sampling). Potential process evaluation participants and carers will then be contacted by phone and invited to participate in a telephone interview. To ensure a range of participants are recruited, variation in sampling will be based on gender, severity of impairment, residential status (living alone/ not alone), study group (control/experimental), and trial site. Patients and carers who agree to an interview will be telephone interviewed by an occupational therapist trained in qualitative interviewing and not involved in delivering trial interventions. At the commencement of the interview, verbal consent to participate with will be audio recorded along with the rest of the interview and sent for professional transcription. Stroke survivor participants and carer interviews will be conducted separately to enable each participant to speak freely about their experience of discharge from rehabilitation and engagement in the trial. The semi-structured interview guide is based on the aims of the trial, process evaluation, and previous process evaluations. The guide was pilot tested and minor changes to the order of questions were made. Semi-structured interviews with approximately 14–16 people with stroke and 10–12 carers from both the experimental and control groups will explore perceptions of discharge planning, transition to home, adherence to self-monitoring, and social support. Additionally, the relationship between the participant and their occupational therapist, perceptions of goal setting and pre and post discharge visits (tailored to study group), sessions with GP involvement (intervention group only) and suggestions for improvement will be explored.

To explore the perspective of trial occupational therapists, focus groups will be held at selected trial sites. Recruitment will aim to obtain data from a sample across the sites with maximum variation in terms of gender, experience, and involvement in the trial. There will be four focus groups with 6–8 therapists in each group and facilitated by an independent researcher. In the focus groups, occupational therapists working within the trial will explore their experiences with discharge planning and the process of delivering the HOME intervention including training, being involved in the trial, implementing the intervention, and the aspects of the trial went well or not so well. Further, adherence to the protocol including implementation barriers and enablers encountered and suggestions for improvement will be explored. Insights gained will provide detailed information to understand the mechanisms of impact of the intervention on the trial outcomes.

To explore ‘adoption’ the process evaluation will describe the perceived acceptance of the HOME intervention by involved occupational therapists and how their attitudes may or may not have shifted after training and involvement in the trial, affecting uptake. A questionnaire will be sent to all trial occupational therapists working in rehabilitation pre-and post-implementation of the intervention. Questions seek to explore occupational therapists’ beliefs about discharge planning and the work they do with inpatient stroke survivors who will be discharged to the community (home environment) at the end of their program. Respondent demographics will be collected and questions will elicit perceptions of discharge planning and patient participation post-discharge. Additionally, pre-discharge occupational therapy home visit practice and beliefs, as well as barriers and enablers of providing discharge planning support to stroke survivors during rehabilitation will be explored. The second questionnaire distributed at the end of the trial will contain additional questions about the HOME trial, including experiences and satisfaction with the trial, perceived impacts, benefits and challenges, and any site-specific issues. The survey is based on the objectives of the trial and process evaluation, as well as previous work. Responses will be rated on a 6-point categorial scale of agreement and obtained through short answer questions. A printed and electronic copy of the questionnaire will be provided to all trial occupational therapists to maximize response rates. Analysis will focus on both perceived acceptance of the intervention and any shifts in beliefs that occur after training and involvement in the HOME Rehab trial.

The ‘implementation’ dimension of the process evaluation aims to describe the HOME rehabilitation intervention as delivered in terms of quality, quantity, adaptations and variations (planned and unplanned). To assess program fidelity to the protocolized intervention and other factors that may impact the outcome, we will audit the trial documentation, training records, fidelity scoring records, and trial protocol variation sheets. Using descriptive statistics to report the findings, these audits will provide insights into how the planned intervention was implemented.

To explore the ‘maintenance’ dimension, we aim to examine the long-term individual and organizational impacts of the intervention and understand the extent to which its delivery was normalized among the healthcare professionals and related practice staff. Twelve months post completion of the trial, the lead investigator will survey each site principal investigator to understand which, if any, elements of the HOME Rehab intervention are used, and if so, how they are integrated into usual discharge planning practices at the site.

### 2.4 Patient and public involvement

Collaborative engagement with stroke survivors, clinicians and policymakers has ensured consumer and community involvement in the design of this process evaluation. The HOME Rehab trial is supported by an end-user advisory panel, inclusive of advisors living with stroke, carers, occupational therapists, health managers and policymakers, who meet on a regular basis; this panel has reviewed all participant-facing documents and will be invited to review emergent themes to ensure involvement through to dissemination. All advisory panel members are paid an honorarium.

### 2.5 Data analysis

#### 2.5.1 Quantitative data

Quantitative data from clinician surveys, trial logs and fidelity checklists will be entered into a password protected database and will be analysed in SPSS using appropriate descriptive and inferential statistics. Analysis will focus on variability across groups, while the extent to which the intervention is delivered as intended will be examined by exploring the proportion of the essential components which was reported as delivered. Variability in the extent to which the HOME Rehab intervention is delivered as intended across sites and change across the duration of the trial will also be examined. Program reach will be assessed by examining the proportion of inpatient with stroke who are admitted to recruiting rehabilitation centers and who are recruited to, and engage with, the HOME Rehab trial.

#### 2.5.2 Qualitative data

For qualitative data analysis interview transcripts will be read several times in their entirety independently by two persons. Codes will be developed based on the conceptual framework offered by RE-AIM, with additional thematic findings identified inductively through the data added to the coding framework. Data will be coded within NVivo 12 (QSR International, Doncaster) and thematically analysed using thematic analysis (22). Potential themes will then be reviewed and refined based on their response and relevance to the research questions. Final themes and subthemes will be determined by discussion between the analysts in consultation with the project team (23). Multiple processes will ensure a reflexive stance throughout data collection and analysis. Regular meetings between the interviewer, a second coder (to ensure nuanced and insightful code development) and two project team members experienced in qualitative analysis will hold reflexive discussions of data interpretation, and a range of perspectives. Enhancing trustworthiness of the analysis, discussion will occur about how researcher assumptions may affect the analysis and examination of alternative interpretations and explanations will be undertaken (24). Strengthening dependability and credibility of the analysis, regular project meetings and presentation of potential themes to peers and stakeholders will ensure the themes reflect a convincing account of the dataset (24). A reflexive journal and detailed records will be kept to document a transparent decision trail of analytical and methodological decision making and data interpretation (25). Further, the dataset will be examined for disconfirming evidence that did not support interpretations (24).

#### 2.5.3 Synthesizing data

The integration of findings from multiple data sources will be an important step in refining the interpretation of outcomes, understanding the context of program delivery, and identifying key findings. Results from the qualitative and quantitative methods will be interpreted together using the RE-AIM framework, and areas of convergence or divergence in the findings noted (21). Integrating the findings from multiple perspectives and methods will strengthen the overall findings and provided detailed insights into the causal mechanisms (as proposed in the logic model Figure 1).

## 3 Discussion

The HOME Rehab trial has been designed to address known challenges with transitioning from an inpatient rehabilitation center to the home environment, and this proposed process evaluation will examine these causal mechanisms as well as the contextual factors that may influence intervention outcomes. The proposed method will provide opportunity to understand the complexity of the HOME Rehab intervention in an iterative manner, while offering a structure to this process so as to ensure past criticisms of process evaluations whereby process evaluations that are conducted ad-hoc or appear to be an afterthought (26) are addressed. Designing the HOME Rehab trial process evaluation as a nested study has ensured that the causal assumptions underpinning the intervention can be tested, and that findings will identify what elements work, when, and in what context (4, 5). We acknowledge that within complex intervention trials, an all-encompassing process evaluation is not possible, instead this process evaluation protocol has identified the key uncertainties held about the interventions and responses to and interactions with the interventions. A potential limitation of this pragmatic approach is that aspects which do not have a substantial influence on the HOME Rehab intervention may have been missed. However, monitoring and evaluating the processes and procedures involved in conducting the trial will enable timely feedback and opportunities for improvement that can help to optimize the study’s outcomes and impact in real-time (6). Additionally, knowledge about how an intervention is implemented, its mechanisms of impact, and the contextual factors that impact on the intervention, provides details for scale up and replication in different settings should the program be effective (5).

### 3.1 Trial status

The HOME trial commenced in 2017, but has encountered multiple delays with restrictions related to the COVID-19 pandemic. Recruitment is ongoing and the trial is expected to be completed in 2024, with final data collection completed in 2025. The process evaluation is embedded within the trial, and therefore conducted across the same time-period.

## 4 Ethics and dissemination

This study received ethics approval from the Alfred Human Research Ethics Committee under the Australian National Mutual Acceptance Scheme (NMA17/236) and site-specific ethics approval has been obtained at all participating sites. Separate participant information and consent forms were signed for all process evaluation interviews by participants (stroke survivors, carers, stroke clinicians and trial occupational therapists).

Results of the process evaluation will be submitted for publication in peer-reviewed journals and presented at selected, relevant conferences. Use of the RE-AIM framework will provide a comprehensive approach to the process evaluation and the expected outputs and outcomes from the trial and process evaluation are shown in the logic model of the HOME Trial (Figure 2).

Understanding the extent the complex HOME rehabilitation intervention was implemented as intended will support interpretation of the outcomes, and make transparent the factors that impacted on its implementation. Further, knowledge of contextual factors, barriers, as well as which elements of the intervention are perceived to be most useful has important implications for effectively delivering comprehensive discharge planning and support after stroke. Knowing the mechanisms of impact that lead to the final outcomes will enable the intervention to be effectively adapted (if necessary), scaled-up (if effective) and applied in practice with appropriate policies. Trial training materials will be shared electronically with participating sites, and following trial completion, will be made available on request.
